# HelixComplex snail mucus as a potential technology against O_3_ induced skin damage

**DOI:** 10.1371/journal.pone.0229613

**Published:** 2020-02-21

**Authors:** Valentina Gentili, Daria Bortolotti, Mascia Benedusi, Andrea Alogna, Anna Fantinati, Anna Guiotto, Giulia Turrin, Carlo Cervellati, Claudio Trapella, Roberta Rizzo, Giuseppe Valacchi

**Affiliations:** 1 Department of Chemical and Pharmaceutical Sciences, University of Ferrara, Ferrara, Italy; 2 Department of Biomedical and Specialist Surgical Sciences, Section of Medical Biochemistry, Molecular Biology and Genetics, University of Ferrara, Ferrara, Italy; 3 Department of Animal Science, Plants for Human Health Institute, North Carolina Research Campus, Kannapolis, North Carolina, United States of America; 4 Department of Food and Nutrition; Kyung Hee University, Seoul, South Korea; School of Pharmacy, Ardabil University of Medical Sciences, ISLAMIC REPUBLIC OF IRAN

## Abstract

Mucus form *H*. *aspersa muller* has been reported to have several therapeutic proprieties, such as antimicrobial activity, skin protection and wound repair. In this study, we have analyzed *H*. *aspersa* mucus (Helixcomplex) bio-adhesive efficacy and its defensive properties against the ozone (O_3_) (0.5 ppm for 2 hours) exposure in human keratinocytes and reconstructed human epidermis models. Cytotoxicity, tissue morphology and cytokine levels were determined. We confirmed HelixComplex regenerative and bio-adhesive properties, the latter possibly via the characteristic mucopolysaccharide composition. In addition, HelixComplex was able to protect from O_3_ exposure by preventing oxidative damage and the consequent pro-inflammatory response in both 2D and 3D models. Based on this study, it is possible to suggest HelixComplex as a potentially new protective technology against pollution induced skin damage.

## Introduction

Being the skin our first defense against the external world, this organ is continuously exposed to several stressors among which pollution has been shown to be among the most toxic [1–3]. Although the troposphere is characterized by the presence of multiple pollutants, O_3_ has been shown to be one of the most toxic and recent evidences have supported the idea that O_3_ is able to not only affect skin homeostasis but also play a role in the development of several skin conditions. Indeed, in the last decade several studies have shown the correlation between O_3_ levels and ER visits for skin diseases [4–6]. Xu et al were able to link skin conditions such as eczema, urticaria, rash/eruption, contact dermatitis, and infection to high 8-hour concentrations of O_3_ [6]. Medical examination for conjunctivitis and skin rash were associated with O_3_ concentrations in a study from 22 cities in France [7]; and we have found positive associations of short-term O_3_ concentrations with hospital admissions for skin conditions (such as cellulitis, dermatitis, urticaria) in multiple areas in Canada [8].

Although it has been shown the ability of O_3_ to induce oxidative damage, O_3_ is not a radical per se and it is too reactive to penetrate the skin. It has now well documented that its ability to affect cutaneous tissues is mainly a consequence of its reaction with the skin lipids present in the stratum corneum leading to the formation of reactive biomolecules among which H_2_O_2_ and aldehydes are among the most reactive [9]. Several natural and synthetic compounds, have been analyzed in the cosmeceutical field to prevent the O_3_ damage to skin [10]. Recently, a growing literature and interest has highlighted the ability of snail secretion (snail mucus), extracted from snails maintained in a laboratory setting. to improve skin conditions thanks to its emollient, moisturizing, lubricating and protective properties [11].

In particular, mucus form *H*. *aspersa muller* has already been reported to have different properties, such as antimicrobial activity [12] and wound repair [13, 14]. The biochemical analysis of *H*. *aspersa* mucus showed the presence of mucopolysaccharide that permits considerable hydrogen bonding with adjacent water molecules, which effectively leads to hydration of the surrounding tissue [13]. In addition, it stimulates endogenous hyaluronate synthesis, resulting in an increase in water-binding capacity and viscoelasticity of the skin [13, 14]. Moreover, the presence of mucopolysaccharide could improve the adhesion of the mucus to the skin and act as a barrier to prevent epithelial cell insults from pollution and the presence of polyphenols could give to the mucus the ability to prevent and counteract the pollution induced cutaneous oxidative damage.

In this study, we have investigated the protective effect of *H*. *aspersa* mucus (HelixComplex) in ozone induced skin damage by the use of both 2D and 3D skin models. Our study supports the topical usage of *H*. *aspersa* mucus (HelixComplex) as a new antipollution technology to prevent premature skin aging.

## Materials and methods

### HelixComplex collection and microbiological evaluation

The *Helix aspersa* snails were fostered in the private snail farming “Corte Frazza” (Via Frattina 22, 44049 Vigarano Mainarda, Ferrara, Italy) (geographical co-ordinates 44°50'37.6"N 11°28'01.3"E), certified for the snail fostering by the local health unit company (AUSL Ferrara) with the permission number 022FE022. *Helix aspersa* mucus (HelixComplex) was collected by HelixPharma industries (Ferrara, Italy), certified for the collection of snail mucus by the local health unit company (AUSL Ferrara) with the permission number ABP5076. The snail mucus was extracted for the purpose of this study using a patented extractor machine (Beatrix^®^; HelixPharma industries; Ferrara, Italy) (Patent N WO2013011371A1) that collects about 600 ml of crude extract from 500 snails (about 10 kg) after 45 minutes. The mucus was obtained using low concentrations of NaCl (3%) that was sprayed on snails. The stress caused by this solution induced the snails to produce mucus, that was collected in underlying canisters. Then the snails were rehydrated and re-entered the field. The process does not cause mortality to the snails, as confirmed by the permission obtained by the local health unit company (AUSL Ferrara) (N: ABP5076). Mucus was then sterilized with a peristaltic pump and a filtration device (0.2μm; Pall) (Patent N 10207000117547), specifically developed for mucus filtration and stored at 4°C or -80°C. The mucus is available by request to HelixPharma industries. The composition of HelixComplex is reported in Table 1 [13].

**Table 1:** Quali-quantitative chemical and microbiological composition of HelixComplex.

Specification	Values	Measure unit
Aspect	Clear	
Color	Yellow	
Smell	Odourless	
pH	7.0	
Density	1.1	
Dry residual	3.2	g/L
Yield%	0.12	
Minerals	350	mg/L
Heavy metals	Absent	
Proteins	250	mg/L
GAGs (sulfurated)	90	mg/L
GAGs (unsulfurated)	80	mg/L
Glycolic acid	<200	mg/L
Allantoin	<20	mg/L
Poliphenols	80	mg/L
Sugars	0.027	g/L
Collagen	80	mg/L
Gram +	0	CFU
Gram −	0	CFU
Fungi	0	CFU

### Cell lines

Human keratinocyte cell line (HaCaT; AddexBio Ca, USA, Catalog N. T0020001) (Certificate of analysis in Supplementary material; S1 Fig) was cultured in DMEM medium (Gibco, Grand Island, NY, USA), supplemented with 1% glutamine, 1% penicillin/streptomycin and 10% FBS. The cells were grown with the 5% CO2 at 37°C as previously described [15].

### 3D skin tissue models

EpiDermTM Tissue Model (MatTek *In Vitro* Life Science Laboratories, Bratislava, Slovak Republic) were kept at 37°C in a 95% humidified, 5% CO_2_ atmosphere in a maintenance medium provided by manufacturers until the exposure. Prior to HelixComplex treatment, media was aspirated and fresh media was added. Reconstructed human epidermis (RHE) was topically treated with or without HelixComplex (ranging from 4 to 400 ug/ml) for 4 hours. Control tissue was exposed to the same doses of vehicle (medium). To avoid excess tissue moistening, a minimum suspension volume was used and tissues were kept at 37°C in a humidified 5% CO_2_ atmosphere in a maintenance medium for 4 hours. The control tissues (with or without HelixComplex treatment) were also exposed to filtered air [16].

### Cell viability and cytotoxicity assays

At different time points after treatment, cell viability was examined by Trypan blue dye exclusion and MTT (3-(4,5-dimethilthiazol-2yl)-2,5-diphenyl tetrazolium bromide) colorimetric assay (Roche Diagnostics Corporation, Indianapolis, IN) as previously described [13].

Cytotoxicity studies were performed after the different treatments by assessing LDH (lactate dehydrogenase) release in the culture media (EuroClone, Milan, Italy). In order to obtain a representative maximal LDH release (100% of toxicity) samples were lysed with 2% (V/V) Triton X-100 in culture media for 30 min at 37°C [17].

### Bioadhesivity test

Bio-adhesivity is an important property of substances presenting the ability to get in close contact with biological structures, allowing a protracted retention period of active molecules. We used an easy and standardized lectin-based assay for evaluation of HelixComplex bioadhesive properties [18]. Lectin is known to be able to bind mucines expressed on mucosal surfaces. For this reason, this assay tested the ability of substances to bind the mucosa and consequently interfere with lectin-mucin interaction. Briefly, cells were seeded in a 8-chamber slide at the final concentration of 20.000/well. After 24 hours, the medium was removed and cells dried for 15 minutes. Cells were fixed with 100 μl of 1:1 methanol/acetone solution for 30 minutes at -20°C and dry again at room temperature. Cells were finally rehydrated for 5 minutes by adding 500 μl of PBS 1x. 200 μl of HelixComplex were added to cells. 200 μl of PBS 1x were used as negative control while 200 μl of a bio-adhesive solution, containing 1g/10ml of Sucralfate gel, diluted 1:5 in sterile water and a solution of 0,8mg/ml of a natural molecular complex containing polysaccharides, natural mineral and Arabic gum were added as positive control. After 15 minutes at 37°C, 200 μl of biotinylated lectin 10 μg/ml was added to each well and let 30 minutes at 37°C. Then, each well was washed three times with 500 μl of 0.05% tween20-PBS 1X for 5 minutes in agitation. After washes, 200 μl of Streptavidine-HRP (2.5 μg/ml) 1:100 diluted, were added for 1 hour at 37°C. Cells were then washed three times and 100 μl of TMB substrate were added in each well for 5 minutes. The reaction was stopped with 100 μl of HCl 1N and 100 μl of sample were transferred on a 96-well plate and read at 450 nm. The absorbance is inversely correlated with bio-adhesive properties of the substance tested.

### In vitro scratch wound assay

The healing properties of HelixComplex were tested on HaCaT cells by scratch assay. Briefly, keratinocytes were seeded at the final concentration of 1x10^6^ in a 6-well plate. After 24 hours, medium was removed and a linear scratch in the middle of the well was done using a p200 tip as previously described [19]. Then 400μl of HelixComplex or media (control) were added to each well. Scratch repair was monitored by optical microscopy.

### Ozone treatment

O_3_ was generated from O_2_ by electrical corona arc discharge (ECO_3_ model CUV-01, Torino, Italy), as previously described [20]. The O_2_–O_3_ mixture (95% O_2_, 5%O_3_) was combined with ambient air and allowed to flow into a Teflon-lined exposure chamber, with the O_3_ concentration in chamber adjusted to 0.5 ppm and continuously monitored by an O_3_ detector. Temperature and humidity were monitored during exposures (37°C and 45–55%, respectively). The control tissues (with or without HelixComplex treatment) were also exposed to filtered air in similar exposure chambers except that filtered airflow was released into the chamber at flow rates similar to the O_3_ output. To assess the efficacy of HelixComplex to protect skin tissues, RHE were pre-treated with HelixComplex 400 μg/ml for 4 hours and then exposed to 0.5 ppm of ozone for 2 hrs (S2 Fig), after 24 the following analysis were performed: i) cell cytotoxicity (LDH assay); ii) tissue morphology by hematoxilin-eosin staining (H&E); iii) H_2_O_2_ levels; iv) 4HNE formation); v) cytokine expression and release.

### Histological analysis

RHE tissues, with or without HelixComplex treatment, exposed to O_3_, were immersion-fixed in 10% NBF (neutral-buffered formalin) for 24 hours at room temperature, then dehydrated in alcohol gradients and embedded in paraffin. For histological observation, the sections (6 μm thickness) were deparafinized in xylene and rehydrated in alcohol gradients (100%, 90%, 80% 70%). The sections were stained with hematoxylin for 3–5 minutes, washed in running tap water no more than 5 min, and then stained with Eosin Y for 2 min. The sections were washed in tap water for 1–5 min, dehydrated in increasing concentrations of alcohols and cleared in xylene. The sections were then mounted with a rapid non aqueous mounting medium, contains tolulene (Entellan, Merck KGaA, Darmstadt, Germany) and observed under Nikon Microphot FXA microscope (Nikon Instruments, Amsterdam, Netherlands) [21].

### RNA extraction

RNA was extracted from RHE using the RNeasy mini kit (Qiagen, Hilden, Germany). DNA contamination in RNA preparations was eliminated by digestion with DNase I RNase-free (Thermo Fisher Scientific, Waltham, MA, USA) and subsequent purification using the MinElute Cleanup Kit (Qiagen, Hilden, Germany). DNA elimination was assured by β-actin PCR amplification prior to reverse transcription, as previously described [22]. RNAs were reverse-transcribed using SuperScript II First-Strand Synthesis System according to the manufacturer’s protocol (Invitrogen Carlsbad, CA, USA). cDNA aliquots corresponding to 200 ng RNA were used for human cytokines expression analysis.

### qPCR

cDNAs were amplified by specific oligonucleotide primers for: IL-1alpha, IL-1beta, IL-6, IL-8, IL-10 and TNF-alpha (Table 2), and RNaseP (Thermo Fisher Scientific, Waltham, MA, USA) was used as house-keeping control gene. qPCR reactions were carried out in triplicate on a QuantStudio3 PCR System using PowerUP SYBR Green Master Mix (Thermo Fisher Scientific, Waltham, MA, USA). Comparative ΔΔCt method was used to evaluate the relative expression of each gene. Fold changes in the expression of cytokines was calculated as 2^−ΔΔCt^.

**Table 2:** Sequences of forward and reverse primers used to amplify the cytokines.

TARGET	FORWARD PRIMER	REVERSE PRIMER
IL-1 alpha	GGAGCTTGTCACCCCAAACT	TCCGAAGTCAAGGGGCTAGA
IL-1 beta	CTGAGCTCGCCAGTGAAATG	TGTCCATGGCCACAACAACT
IL-6	TAGGACTGGAGATGTCTGAGGCT	GACCGAAGGCGCTTGTGGA
IL-8	GGTGCAGTTTTGCCAAGGAG	TTCCTTGGGGTCCAGACAGA
IL-10	GCTGGAGGACTTTAAGGGTTAC	GATGTCTGGGTCTTGGTTCTC
TNF-alpha	TGGCGTCTGAGGGTTGTTTT	CACCAAGGAAGTTTTCCGCTG

### Hydrogen peroxide (H_2_O_2_) analysis

H_2_O_2_ level was measured by Amplex Red Hydrogen Peroxide/Peroxidase assay kit (Life Technologies) [23]. The quantity of H_2_O_2_ was determined by comparing its absorbance with that of a H_2_O_2_ standard curve according to the manufacturer’s instructions. Resorfurin formation due to Amplex Red (25 μM) oxidation by HRP (0.5 U/ml) bound to H_2_O_2_, was measured in Synergy H1 Hybrid Multi-Mode Reader (BioTek Instruments, Inc., Winooski, VT, US) at 530 nm (excitation) and 590 nm (emission). After an initial stabilization period, 10 μl of maintenance medium were added to the reaction mixture. A calibration curve was performed using H_2_O_2_ solutions as standard and its production level was expressed in nM. Controls in the absence of sample or HRP indicate that nonspecific probe oxidation was minimum (< 1%). Results were expressed as nmol/min mg protein.

### 4-hydroxynonenal (4-HNE) levels

RHE 4-hydroxynonenal (4-HNE) levels before and after O_3_ exposure in presence or not of HelixComplex, were evaluated by commercially available kit (BioSource, Milan, Italy). The measured amount of 4-HNE protein adduct was normalized with protein concentration measured with the Bradford method. A calibration curve was performed using 4-HNE standard. Results are expressed as μg 4-HNE/mg protein.

### Cytokine analysis

The levels of IL-6 and IL-10 were tested in culture supernatants by ELISA assay (Mybiosource, CA, USA) in all the different samples combinations. The ranges of detection were 469pg/ml-300pg/ml and 7.813-500pg/ml for IL-6 and IL-10, respectively. The assay sensitivity was <0.3pg/ml for IL-6 and 4.688pg/ml for IL-10.

### Statistics

Results were expressed as mean value ± SEM and represent the mean of triplicate determinations obtained in 3 separate experiments. Mann Whitney U-test was used to determine statistical significance (p values < 0.05 were considered significant).

## Results

### Evaluation of HelixComplex cytotoxicity

**Fig 1:**
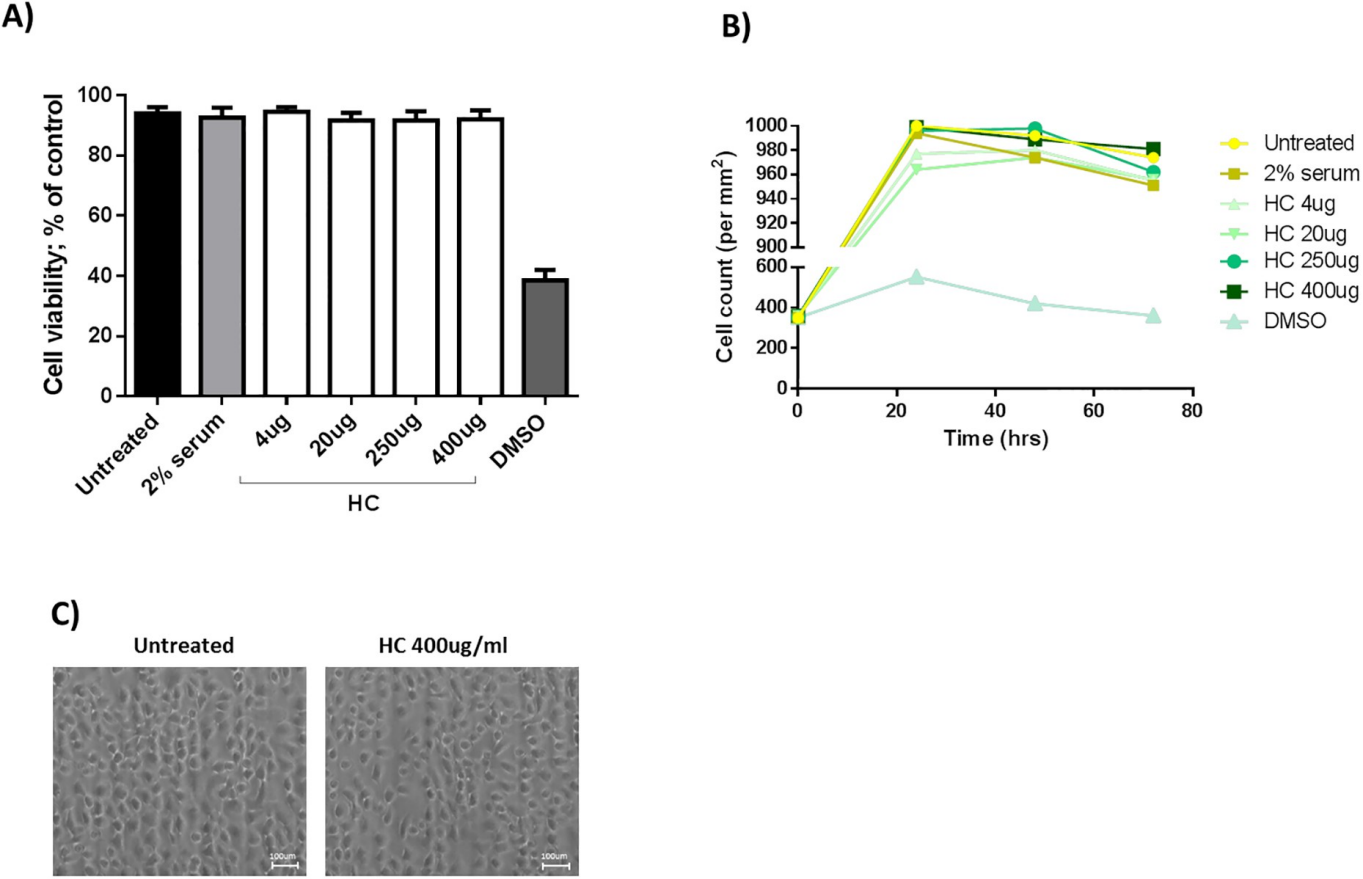
Evaluation of HelixComplex cytotoxicity. Keratinocytes were exposed to increasing doses of HelixComplex (HC) for up to 72 hours and cell viability was examined by MTT colorimetric assay. A) Cell viability was calculated at 24, 48 and 72 hours as percentage with respect to the control untreated cultures (set to 100% for each cell line). DMSO was used as positive control of cell death. B) Cell number was monitored over time by Trypan Blue staining. C) Representative images taken by light microscopy of monolayers of keratinocytes untreated or treated with HelixComplex at 48 hours. Magnification 100X.

The first step of our study was to evaluate the cytotoxicity of the HelixComplex in human keratinocytes. Cells were treated with a range of concentrations (4–400μg/ml) of HelixComplex, and both LDH release (24hr) and morphology were assessed. As shown in Fig 1A and 1B, the treatment with HelixComplex did not shown any toxic effect at the doses tested. DMSO 10% was used as a positive control. In addition, treated keratinocytes with 400μg/ml of HelixComplex showed a normal morphology (Fig 1C).

### HelixComplex improves “in vitro” wound repair

**Fig 2:**
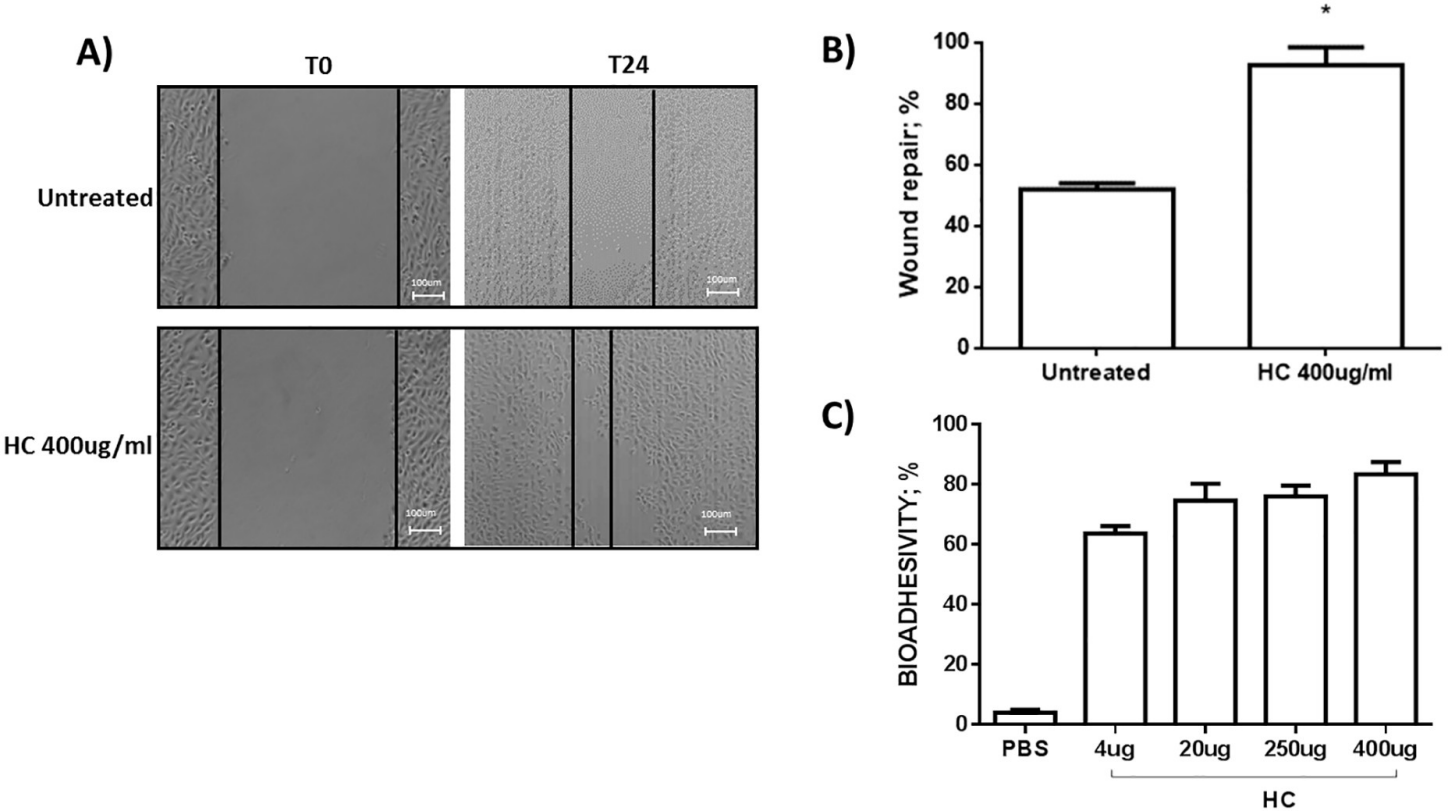
Scratch-wound healing assay and bio-adhesivity. **A**) scratch-wound healing assay, with representative images taken at the indicated time points post wounding; **B)** quantification of wound repair at 24 hours expressed as % of wound repair in comparison with the 0 hour time point (*p = 0.0003); **C)** Bio-adhesivity of HelixComplex on human keratinocytes assessed by a lectin-based assay.

Beside the cell viability, other important markers of keratinocytes physiological responses are their migration and proliferation properties that can be tested by the scratch wound assay. As shown in Fig 2A, pre-treatment with HelixComplex was able to improve the “in vitro” wound healing process after 24 hours respect to the control, where only 50% of the wound area was recovered (p = 0.0003) (Fig 2B).

### HelixComplex presents bio-adhesive properties on human keratinocytes

The efficacy of a skin treatment might be improved by the compound bio-adhesive properties; therefore we assessed the bio-adhesive rate of HelixComplex on human keratinocytes. As showed in Fig 2C, HelixComplex at the concentration of 400 μg/ml significantly prevent lectin binding to the keratinocytes surface (p = 0.0036), with a bio-adhesive property of about 80%.

### Effect of HelixComplex pre-treatment in ozone induced tissue damage

**Fig 3:**
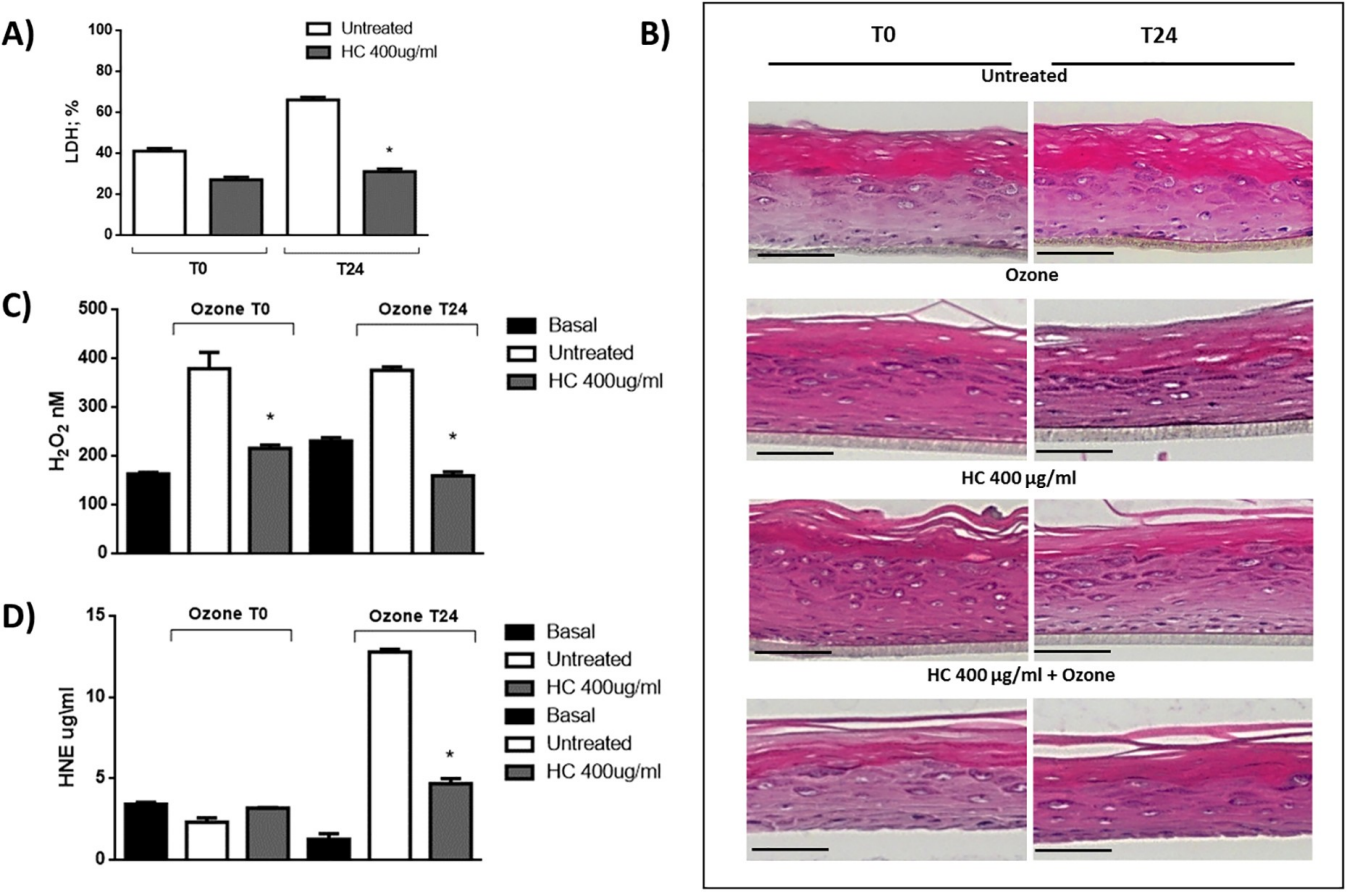
Effect of HelixComplex pretreatment in ozone induce tissue damage. A) Cytotoxicity evaluation was performed by LDH quantification. (*p = 0.012); B) Tissue morphology was evaluated by H&E staining. Basal, spinous, granulous and cornified epidermal layers are represented. Magnification 40X; Nikon Microphot FXA microscope (Nikon Instruments); C) The ability of O_3_ to induce oxidative stress was evaluated by H_2_O_2_ levels in RHE exposed to O_3_ and pre-treated with HelixComplex. (*T0 p = 0.02; T24 p = 0.0013); D) 4-Hydroxynonenal (4-HNE) levels (*T24 p = 0.001).

Tissue damage was evaluated by LDH release and as expected, right after O_3_ exposure (T0) there was an increase levels of LDH of circa 40% that further increased at the later time point to 67% (T24). On the other hand, pretreatment with HelixComplex significantly prevented LDH release from the tissue after O_3_ at T0 and T24 (28% and 30% respectively). (Fig 3A) (p = 0.012).

To evaluate the possible effects of HelixComplex on tissue morphology, H&E staining was performed on 3D skin tissues. As shown in Fig 3B, H&E histology reveals an intact structure of the epidermis where all the epidermis layers are clearly represented (basal, spinous, granulous and cornified epidermal layers). Respect to the control tissues, the HelixComplex treatment did not affect the RHE morphology over the experimental procedure. In particular, the stratum basale, containing mitotically active cells, was comparable in treated or non-treated tissues with a single layer of cuboidal-shaped cells. Concerning the stratum spinosum, the number of cell layers were similar in treated and non-treated samples and the characteristic stratum granulosum, with its grainy appearance and three to five layers of cells was analogues in all experimental conditions. Finally, the stratum corneum, the most superficial layer of the epidermis, did not show noticeable differences in the HelixComplex treated or non-treated tissues. As expected, while cytotoxicity was observed immediately after ozone exposure, the tissue morphology it has not been altered by a single pollutant insult.

### H_2_O_2_ and 4-HNE levels in RHE pretreated with HelixComplex

As mentioned previously, ozone damage is maily mediate by the formation of bioactive molecules among which H_2_O_2_ and 4-HNE. The Amplex Red-Horseradish Peroxidase (HRP) method confirmed that the exposure to ozone increased the levels of H_2_O_2_, while the pre-treatment with 400 μg/ml of HelixComplex for 4 hours prevented is formation at both time points (T0 and T24) as depicted in Fig 3C.

Parallel with the O_3_-induced increase in H_2_O_2_in the medium, we observed an increase in intracellular levels of 4-Hydroxynonenal (4-HNE). Also in this case the increased levels of 4HNE protein adducts (T24) were kept at the baseline levels by the pre-treatment with HelixComplex (Fig 3D).

### HelixComplex prevents ozone induced tissue pro-inflammatory status

A well-documented connection of altered redox homeostasis is with the inflammatory process.

**Fig 4:**
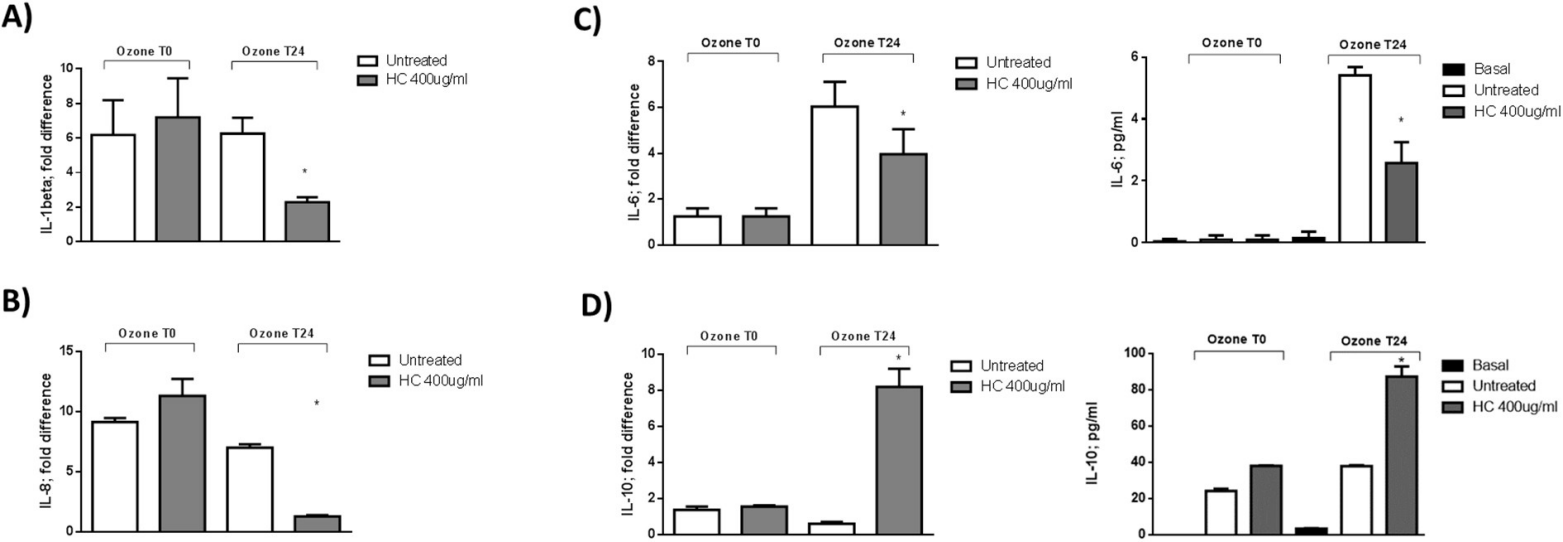
Induction of molecular signals by HelixComplex treatment on RHE model. A) IL-1beta mRNAs levels (*p = 0.0011), B) IL-8 (*p<0.001) mRNAs levels, C) IL-6 (*p = 0.0024) mRNAs (left panel) and protein (right panel) D) IL-10 (*p<0.0001) mRNAs (left panel) and protein (right panel). IL-6 (*p<0.001) and IL-10 (*p<0.0001) protein levels were determined by ELISA assay.

The induction of inflammation was evaluated by the analysis of Th1 (IL-1 beta; IL-6; IL-8) and Th2 (IL-10) cytokines. As shown in Fig 4, after 24hr of exposure, HelixComplex pre-treatment was able to prevent ozone induced increase of both IL-1beta and IL-8 mRNAs (Fig 4A and 4B). IL-6 mRNA was 6 times higher at T24 after O_3_ exposure and the pre-treatment with HelixComplex partially prevented (50%) its expression (Fig 4C) at both mRNA (left panel) and protein (right panel) levels (p = 0.01; p<0.001, respectively). IL-10 mRNA expression was not significantly modified by O_3_ treatment, although the pre-treatment with HelixComplex induced an increase of its expression at T24 (Fig 4D) at both mRNA (left panel) and protein (right panel) levels (p<0.001; p = 0.012, respectively, Student t test).

## Discussion

The recent report of the European Environmental Agency showed that up to 30% of the urban population in the EU is exposed to O_3_ concentrations above the EU limit, and circa 98% to concentrations above the WHO recommendations [24]. This is a constant threat for living organisms since O_3_ is one of the most toxic compounds to which they are continuously exposed [25]. Therefore, the definition of new technologies to counteract the toxic effect of O_3_ to skin would be of extreme help especially for the population living in polluted urban areas.

In the present study we have tested the efficiency of HelixComplex, the purified mucus from *Helix aspersa muller*, as bio-adhesive, regenerative and protective agent against O_3_ toxicity. Our study was able to demonstrate that HelixComplex treatment did not affect the morphology nor the cell viability of the models used in this study. On the other hand, our data suggest the ability of HelixComplex to improve “in vitro” wound healing, suggesting possible regenerative properties. These data confirm previous work where similar characteristics have been observed in skin fibroblasts. Because of the complex chemical composition of the HelixComplex [13], at this stage it is not possible to understand which one is the molecule more responsible for these beneficial properties. A recent study has compared the effect of HelixComplex with allantoin and glycolic acid which are among the main compounds present in HelixComplex. The results have shown that both allantoin and glycolic acid alone did not have any regenerative properties, suggesting that possibly the beneficial effect is a consequence not only of all the molecules present in the mixture but also of the specific ratio of which each component is present in the natural mucus. In addition, as reported previously, HelixComplex composition is also characterized by the presence of polyphenols that, although the exact type of polyphenols is not yet identified, it has been well recognized their ability to quench, either directly or indirectly, pro-oxidant molecules [26]. O_3_ is a small molecule and has a strong oxidizing property that can act directly on the surface of the skin affecting the deeper layers of the cutaneous tissues via the generation of a cascade of bioactive compounds. Indeed, although it is not a radical species *per se*, O_3_ is able to oxidize components of the cell membrane, mainly lipids, generating classical radical species such as hydroxyl radicals that, in turn, drive the production of cytotoxic, non-radical species including aldehydes [27]. It should be mentioned that O_3_ is not able to penetrate the tissue and its action is completely consumed in the outmost layer of the skin, the stratum corneum (SC) which is rich in fatty acids, cholesterol and ceramide, all molecules easily prone to be oxidized. It has been shown that once O_3_ interacts with the SC is able to generate the formation of secondary messengers able to trigger signaling cascades across the different layers of skin, leading to pro-oxidative and inflammatory processes [28]. For this reason, in our study we have used 3D skin model, to mimic the interaction that ozone has with cutaneous tissues. Indeed, this model is characterized by the presence of all the epidermis layers including the stratum corneum and is one of the most reliable model to study cutaneous toxicology [29]. Previous and recent works have shown that ozone exposure was able to increase levels of 4-HNE in human skin confirming the concept previously advanced by Pryor et al. [30–32] in relation to the respiratory tract. According to this hypothesis, the exposure of non-cellular constituents of surface epithelial cells to O_3_ is capable of generating toxic peroxidation products among which 4-HNE and H_2_O_2_ seem to be the most responsible for ozone toxicity [33].

This led us determine the levels of both 4-HNE and H_2_O_2_ in our system and as expected O_3_ exposure clearly increase both molecules. Of note is that pre-treatment with HelixComplex prevented the formation of 4-HNE and H_2_O_2_ suggesting either direct or indirect antioxidant property. It is possible that the presence of polyphenols could activate defensive mechanisms such as the NRF2 pathways and this can trigger an antioxidant response able to prevent or quench O_3_-induced tissue redox imbalance [26]. For instance, in our previous work, the combination of different natural molecules such as Ferulic Acid, vitamin E and vitamin C were able to prevent ozone induced oxidative damage in keratinocytes, in RHE and also in human skin via NRF2 activation, suggesting that ozone noxious effect can be modulated by tissue antioxidant responses [10, 32].

Modification of redox homeostasis has been associated with a pro-inflammatory status [34], therefore in our work we have evaluated the cutaneous induction of pro- and anti-inflammatory cytokines upon ozone exposure. As observed in this study, ozone exposure clearly induced the levels of IL-8, IL-6 and IL-1beta, all cytokines associated to skin inflammatory conditions. It should be mentioned that all those pro-inflammatory mediators have NFkB in their promoter, which is a well-known redox sensitive transcriptional factor. Several groups, including ours, were able to clearly show that ozone is able to induce NFkB activation, not only in skin but in other target tissues including lungs [35]. Therefore, it is possible that the formation of pro-oxidant molecules such as H_2_O_2_ lead to the activation of NFkB which then will transcribe for pro-inflammatory mediators that will contribute to an inflammatory skin condition [36]. Of note, pre-treatment with HelixComplex was able to significantly and clearly prevent the induction of pro-inflammatory cytokines while appeared to induce the anti-inflammatory mediator IL-10, suggesting a clear anti-inflammatory property.

## Conclusion

This study has further evidenced the ability of HelixComplex to counteract ozone-induced skin damage highlighting its future usage as a new anti-pollution topical technology. Whether its beneficial properties can be extended also to other pollutants is still under investigation and in addition, in vivo and human studies need to be performed to better confirm the HelixComplex antipollution properties.

## Supporting information

S1 Fig HaCaT cell line certificate of analysis. (PDF)

S2 Fig 3D models experimental procedure. (TIF)
