# Supplementary material for: HelixComplex snail mucus as a potential technology against O3 induced skin damage
Source: PLoS One. 2020 Feb 21;15(2):e0229613. doi: 10.1371/journal.pone.0229613 (PMC7034816; doi:10.1371/journal.pone.0229613)
Supplement: S1 Fig — (PDF) [file pone.0229613.s001.pdf]

AddexBio
Research Services

4907 Morena Blvd, Ste 1408
San Diego, CA 92117
www.addexbio.com
Tel: (858) 348-7819
customersupport@addexbio.com

Certificate of Analysis: HaCaT Cells

Catalog #: T0020001

Product Information

**Description:**	*In vitro* spontaneously human transformed keratinocytes from histologically normal skin. For Research Use Only, not for in vitro Diagnostic procedures.
**Lot Number:**	0003798
**Storage Conditions:**	Vapor Phase of Liquid Nitrogen

Testing and Results

Test	Result
**Viability by Trypan Blue exclusion test**	>81% viable
**[illegible]phology**	Keratinocyte
**Sterility Test:**	
**Antibiotic-, antimycotic-free culture for detection of bacteria, yeast, and other fungi**	Not detected
**PCR detection of mycoplasma**	Not detected
**PCR detection of HIV-1 virus**	Not detected
**PCR detection of Hepatitis B virus**	Not detected
**PCR detection of Hepatitis C virus**	Not detected
**PCR detection of HPV, EBV, & CMV**	Not detected

Species Determination: STR analysis

Human (DNA Profile)
D5S818: 12
D13S317: 10,12
D7S820: 9,11
D16S539: 9,12
vWA: 16,17
THO1: 9.3
Amelogenin: X
TPOX: 11,12
CSF1PO: 9,11

Signature: Olivia McCole

Date 2014-01-06 10:15:00
Olivia McCole, QC Manager, Quality, Compliance and Bio-safety, email-omccole@addexbio.com

AddexBio hereby represents and warrants that the material provided under this certificate has been subjected to the tests and procedures specified and that the results described. This product is intended to be used for laboratory research use only.
