# Supplementary figures and images for: HelixComplex snail mucus as a potential technology against O3 induced skin damage
Source: PLoS One. 2020 Feb 21;15(2):e0229613. doi: 10.1371/journal.pone.0229613 (PMC7034816; doi:10.1371/journal.pone.0229613)

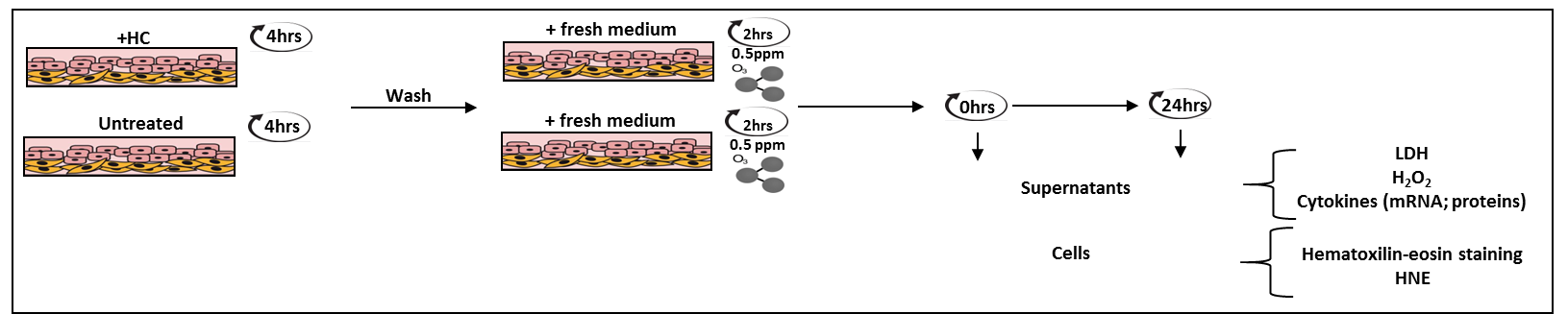

Supplement: S2 Fig — (TIF) [file pone.0229613.s002.tif]
